# Long-Term Outcomes of Anatomical and Biological Borderline Resectable Pancreatic Ductal Adenocarcinoma After Gemcitabine/S-1-Based Neoadjuvant Chemoradiotherapy

**DOI:** 10.3390/cancers18213521

**Published:** 2026-11-02

**Authors:** Haruna Komatsubara, Shugo Mizuno, Daisuke Noguchi, Naohisa Kuriyama, Aoi Hayasaki, Yuki Segi, Kazuyuki Gyoten, Takahiro Ito, Yusuke Iizawa, Takehiro Fujii, Akihiro Tanemura, Yasuhiro Murata, Masashi Kishiwada

**Affiliations:** Department of Hepatobiliary Pancreatic and Transplant Surgery, Mie University Graduate School of Medicine, 2-174 Edobashi, Tsu City 5148507, Mie, Japan; h-komatsu@med.mie-u.ac.jp (H.K.); mizunos@med.mie-u.ac.jp (S.M.); naokun@med.mie-u.ac.jp (N.K.); a-hayasaki@med.mie-u.ac.jp (A.H.); y-segi@med.mie-u.ac.jp (Y.S.); k-gyoten@med.mie-u.ac.jp (K.G.); itotakahiro@med.mie-u.ac.jp (T.I.); uskm007@med.mie-u.ac.jp (Y.I.); t-fujii@med.mie-u.ac.jp (T.F.); iorichan@med.mie-u.ac.jp (A.T.); yasumura@med.mie-u.ac.jp (Y.M.); kishiwad@med.mie-u.ac.jp (M.K.)

**Keywords:** pancreatic ductal adenocarcinoma, borderline resectable, neoadjuvant chemoradiotherapy, long-term outcome, neutrophil-lymphocyte-to-monocyte ratio, systemic inflammation, prognosis

## Abstract

**Background/Objectives:** Oncological outcomes in borderline resectable pancreatic ductal adenocarcinoma (BR-PDAC) remain unclear. This long-term observational study evaluated these outcomes in patients with BR-PDAC treated with a consistent neoadjuvant chemoradiotherapy (CRT) regimen and explored host-related prognostic markers. **Methods:** We retrospectively analyzed 206 patients with PDAC who received neoadjuvant gemcitabine plus S-1 with concurrent radiotherapy between 2011 and 2022. Biological BR was defined as anatomically resectable disease with an initial carbohydrate antigen 19-9 (CA19-9) level ≥500 U/mL. Anatomical BR was subdivided into portal venous involvement (BR-PV) and arterial involvement (BR-A). Outcomes were compared according to resectability and surgical resection, with resectable (R) patients as a reference. Prognostic factors among resected BR patients were assessed using multivariable Cox regression analysis. **Results:** The median follow-up was 62.5 months. Resection rates were 85.7%, 83.3%, 69.8%, and 55.6% for R, biological BR, BR-PV, and BR-A, respectively. Although the resection rate of biological BR was similar to that of R, its survival did not differ significantly from that of anatomical BR. Across all BR subtypes, surgical resection was associated with longer overall survival. Among resected patients, the post-CRT neutrophil-lymphocyte-to-monocyte ratio (NL/M), a peripheral blood cell index, was associated with overall survival (hazard ratio 1.020, *p* = 0.009). Patients with a high post-CRT NL/M had poorer prognosis than those with a low NL/M (*p* = 0.001). **Conclusions:** Biological and anatomical BR-PDAC showed no significant difference in long-term survival after neoadjuvant CRT, and surgical resection was associated with longer survival across BR subtypes. Post-CRT NL/M was associated with overall survival after resection and may serve as a potential host-related prognostic marker, although further validation is needed.

## Simple Summary

Borderline resectable pancreatic ductal adenocarcinoma (BR-PDAC) includes tumors with anatomical or unfavorable biological characteristics that may limit the likelihood of successful curative resection. The present study revealed long-term outcomes in this population treated with a consistent neoadjuvant chemoradiotherapy regimen and explored blood-based markers that might help predict outcomes after treatment. Patients with anatomically resectable tumors but an initially high carbohydrate antigen 19-9 level (≥500 U/mL) were classified as biologically BR. Patients with biologically and anatomically BR disease showed no statistically significant difference in survival, despite a higher resection rate in the biological group. Among patients who underwent surgical resection, a blood cell index measured after chemoradiotherapy, neutrophil-lymphocyte-to-monocyte ratio (NL/M), was associated with survival. Patients with a high NL/M had poorer prognosis than those with a low NL/M. These findings provide fundamental information, including long-term outcomes, on BR-PDAC patients after neoadjuvant chemoradiotherapy.

## 1. Introduction

Pancreatic cancer remains one of the most lethal malignancies, with a 5-year relative survival of approximately 10–15% [1,2,3]. Although treatment outcomes for pancreatic ductal adenocarcinoma (PDAC) have improved with advances in systemic therapy [4,5], the increasing use of neoadjuvant treatment, and improvements in surgical techniques, surgical resection remains the only potentially curative option, and only a limited proportion of patients are candidates for curative-intent resection [6]. In particular, borderline resectable (BR) PDAC represents a heterogeneous disease entity associated with a high risk of margin-positive resection and early recurrence. In addition to anatomical factors, the 2017 international consensus criteria [7] proposed biological and conditional dimensions of borderline resectability, highlighting the importance of tumor biology and patient condition in treatment decision-making.

For anatomical BR-PDAC, involvement of major vessels makes upfront R0 resection challenging. In contrast, biological BR-PDAC is anatomically resectable but is considered to have unfavorable oncological outcomes [8,9,10,11,12]. Neoadjuvant therapy has therefore been increasingly used to provide early treatment of potential micrometastatic disease, increase the likelihood of R0 resection, and allow preoperative assessment [4,13]. However, the optimal neoadjuvant treatment strategy for BR-PDAC has not yet been established, and substantial heterogeneity remains in treatment regimens and surgical strategy [14,15,16,17]. At our institution, neoadjuvant CRT for PDAC was introduced in 2005, and GS-CRT has been consistently used since October 2011. This long-standing, relatively uniform treatment strategy allows us to evaluate long-term oncological outcomes after neoadjuvant CRT in a consistent clinical setting. However, long-term outcomes under a consistent strategy remain limited, particularly when biological borderline resectability is also considered. Such long-term data may provide clinically relevant insights into the future management of BR-PDAC.

To support clinical decision-making, reliable biomarkers that provide information on treatment response and subsequent prognosis are needed. Serum carbohydrate antigen 19-9 (CA19-9) is widely used as a tumor marker in PDAC. Several studies have demonstrated that normalization or substantial reduction in CA19-9 during neoadjuvant therapy is associated with favorable oncological outcomes [18,19], and CA19-9 response has gained importance in determining the timing of surgery. However, the optimal cutoff and a meaningful response remain unclear. Moreover, CA19-9 levels may also be influenced by nonmalignant conditions, including biliary obstruction, cholangitis [20], and Lewis antigen status.

In addition to tumor markers, host-related factors, such as nutritional status [21], systemic inflammation [22,23], and immune response, have increasingly been recognized as potential prognostic indicators in patients with PDAC. Representative indices include the prognostic nutritional index (PNI) [21], neutrophil-to-lymphocyte ratio (NLR) [22], lymphocyte-to-monocyte ratio (LMR) [23], and systemic inflammation response index (SIRI) [24]. We previously reported [21] the prognostic significance of post-CRT PNI in PDAC patients after CRT. That study analyzed all patients who underwent neoadjuvant CRT regardless of resectability, and the PNI stratified outcomes in both resected and unresected patients.

This study aimed to evaluate long-term oncological outcomes in patients with BR-PDAC treated with a consistent neoadjuvant CRT strategy and to explore clinically applicable prognostic markers after CRT, with particular attention to host-related factors.

## 2. Methods

### 2.1. Study Design

**Figure 1:**
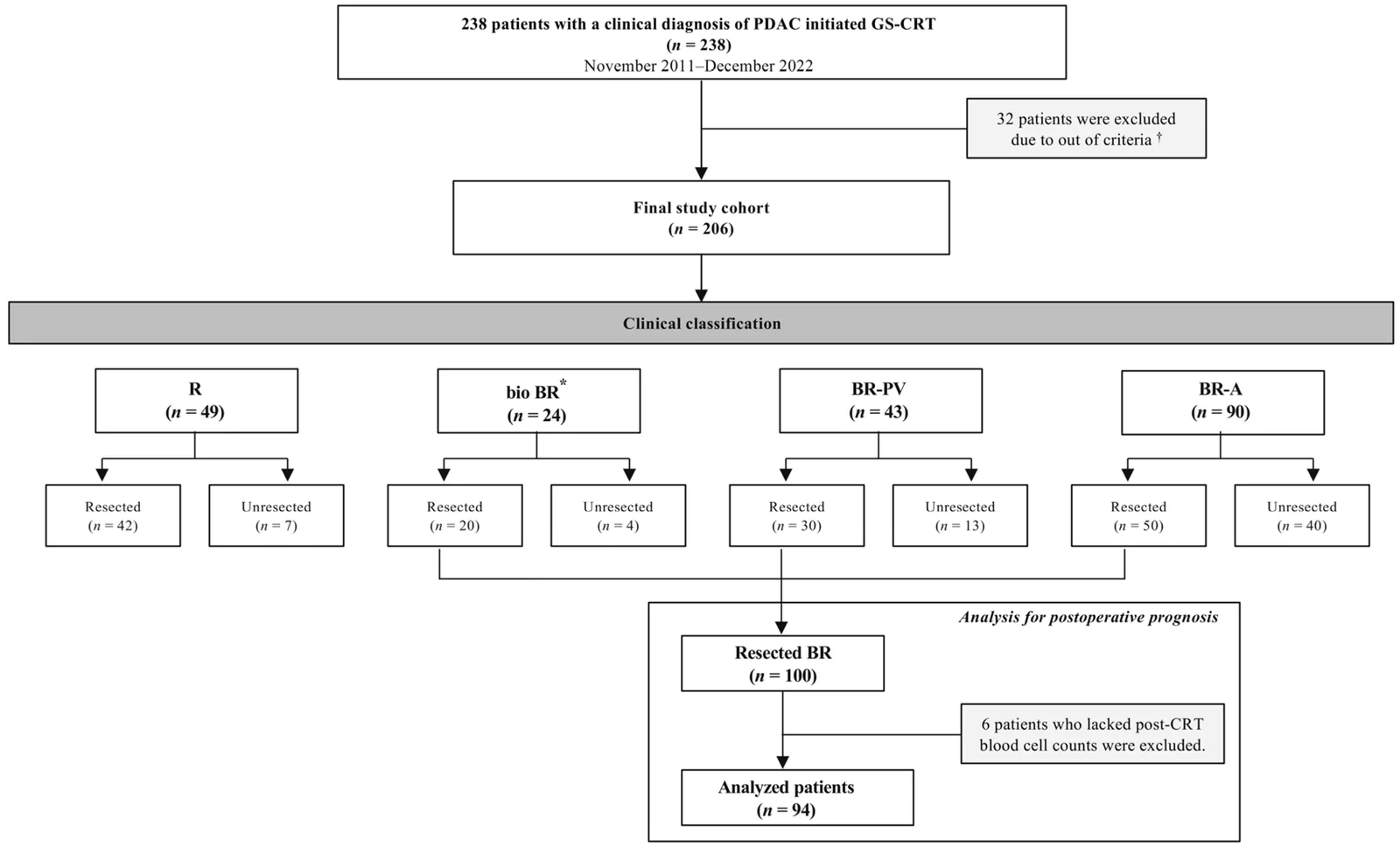
Study flow diagram. Abbreviations: bio BR, biologically borderline resectable; BR-A, borderline resectable with arterial involvement; BR-PV, borderline resectable with portal vein involvement; CRT, chemoradiotherapy; GS-CRT, gemcitabine plus S-1 chemoradiotherapy; PDAC, pancreatic ductal adenocarcinoma; R, resectable. * bio BR was defined as an initial serum CA19-9 level ≥500 U/mL. † Eight patients were excluded because the final diagnosis was not PDAC, including mucinous carcinoma, acinar cell carcinoma, distal cholangiocarcinoma, or benign disease. 24 patients were excluded: 14 who were unable to continue CRT or did not undergo post-treatment reassessment, three who transferred to another hospital during treatment, two with lesions suspicious for distant metastases before treatment that were subsequently confirmed as metastatic disease during treatment or at reassessment, one with liver metastasis who underwent combined pancreatic and hepatic resection, and four who received CRT at another institution.

From November 2011 to December 2022, 238 patients with pancreatic cancer classified as resectable (R) or BR were scheduled to receive neoadjuvant GS-CRT and were assessed for eligibility. Eight patients were excluded because the final diagnosis was not PDAC, including mucinous carcinoma, acinar cell carcinoma, distal cholangiocarcinoma, or benign disease. An additional 24 patients were excluded: 14 who were unable to continue CRT or did not undergo post-treatment reassessment due to reasons such as worsening comorbidities or patient refusal, three who transferred to another hospital during treatment, two with lesions suspicious for distant metastases before treatment that were subsequently confirmed as metastatic disease during treatment or at reassessment, one with liver metastasis who underwent combined pancreatic and hepatic resection, and four who received CRT at another institution. Consequently, 206 patients with R or BR-PDAC were included in the analysis. The detailed patient selection process is shown in Figure 1.

All patients provided written informed consent for inclusion in this study. The study protocol was approved by the Medical Ethics Committee of Mie University Hospital (Approval No. H2020-118) and was conducted in accordance with the ethical standards of the latest version of the Declaration of Helsinki which was revised in 2024.

### 2.2. Definitions of Resectability Categories

Anatomical resectability was classified according to the 7th edition of the Japan Pancreas Society (JPS) classification [25]. Resectable disease was defined as no tumor contact with the superior mesenteric vein/portal vein (SMV/PV), or tumor contact of <180 degree without occlusion, and no tumor contact or invasion of the superior mesenteric artery (SMA), celiac artery (CA), or common hepatic artery (CHA). Biological BR disease was defined based on the concept proposed by the 2016 International Association of Pancreatology (IAP) consensus. Because pathological or positron emission tomography–computed tomography (PET-CT)-based assessment of regional lymph node metastasis was performed only in a limited number of patients at our institution, biological BR was defined using serum CA19-9 levels. This definition is therefore a simplified adaptation of the biological criterion of the international consensus. In patients with obstructive jaundice, CA19-9 levels were assessed after adequate biliary drainage and confirmation of bilirubin reduction to minimize the influence of hyperbilirubinemia. Accordingly, patients with anatomically R disease and a serum CA19-9 level ≥500 U/mL at initial presentation were classified as having biological BR. Lewis antigen status was not routinely assessed, and patients with low or non-elevated CA19-9 levels were not excluded. CA19-9 was generally measured before CRT and at post-CRT reassessment, and changes during CRT were not used to determine biological BR status. BR-PV was defined as tumor contact or invasion of the SMV/PV of ≥180 degree or occlusion, provided that the involvement did not extend beyond the inferior border of the duodenum, with no tumor contact or invasion of the SMA, CA, or CHA. BR-A was defined as tumor contact or invasion of the SMA or CA of <180 degree without stenosis or deformity, or tumor contact or invasion of the CHA without involvement of the proper hepatic artery (PHA) and/or CA. When both the SMV/PV and peripancreatic arteries were involved, the disease was classified as BR-A.

### 2.3. Treatment Detail

The neoadjuvant GS-CRT protocol has been described previously [26,27,28,29,30,31]. As shown in Figure S1, neoadjuvant GS-CRT consisted of concurrent S-1, gemcitabine, and three-dimensional conformal radiotherapy (3D-CRT). S-1 was administered orally at 60 mg/m^2^/day in two divided doses on days 1–21 of a 28-day cycle, and gemcitabine was administered intravenously at 600 mg/m^2^ on days 8 and 22. Radiotherapy was delivered concurrently to a total dose of 50.4 Gy in 25–28 fractions, five fractions per week. The radiation field encompassed the primary tumor, clinically involved regional lymph nodes, and the relevant perivascular regions, with appropriate margins for respiratory motion and setup uncertainty. Treatment planning was performed with consideration of dose constraints for organs at risk, particularly the kidneys and spinal cord. Patients underwent reassessment 4–6 weeks after completion of CRT, and pancreatectomy was considered when curative-intent resection was deemed feasible.

### 2.4. Indication of Surgical Resection

Patients were re-evaluated 4–6 weeks after completion of CRT to determine their eligibility for surgical resection. The decision to proceed with surgery was based on a comprehensive assessment of radiological findings, including changes in the primary tumor and vascular involvement, the absence of distant metastases, changes in serum tumor marker levels, and the patient’s general condition. In patients with R or BR-PV disease, pancreatectomy was considered when curative-intent resection was judged to be technically feasible. For patients initially classified as BR-A, particular attention was paid to the relationship between the tumor and the major arteries at post-CRT imaging. Surgical resection was generally considered in patients showing favorable changes in local tumor extent after CRT; however, the absence of downstaging did not necessarily preclude surgery when dynamic CT showed no encasement or deformity of the celiac artery, superior mesenteric artery, or jejunal arteries. For patients with biological BR disease, surgical resection was considered when the disease remained anatomically resectable after CRT and a decrease in serum CA19-9 was observed, although no predefined threshold for the magnitude of CA19-9 reduction was used. Additional factors considered in the decision to proceed with surgery included the absence of distant metastases and a performance status of 0–1. When these findings collectively indicated that curative-intent resection was feasible, patients proceeded to pancreatectomy.

### 2.5. Surgical Procedure

Following re-evaluation, either pancreaticoduodenectomy (PD), distal pancreatectomy (DP) or total pancreatectomy (TP) was performed depending on tumor location (head, body or tail). SMV/PV resection and subsequent vascular reconstruction were conducted if the surgeon was unable to dissect off the pancreatic head from these vessels without leaving gross tumor on the vessels. Segmental resection and re-anastomosis of the common hepatic artery (CHA) was also performed if tumor involvement was limited to the CHA. Regarding lymph node dissection, no extended lymph node dissection was performed except for regional lymph node dissection as defined by the JPS 7th classification [25]. Intra-operatively, those patients who were found to be unresectable, usually due to the presence of distant metastases, underwent surgical bypass according to clinical indications.

### 2.6. Adjuvant Chemotherapy and Postoperative Follow-Up

Following surgical resection, adjuvant chemotherapy with S-1 was administered whenever feasible. S-1 was given orally for 4 consecutive weeks followed by a 2-week rest period, constituting one 6-week cycle, and treatment was continued for approximately 6 months. After surgery, patients were regularly followed in the outpatient setting. Physical examinations were performed monthly, and blood tests, including measurement of serum CA19-9 levels, were obtained every 2–3 months. MDCT was performed every 3–6 months during the first 2 years after surgery and every 6 months thereafter. In patients who developed recurrence, the site of the first recurrence was recorded.

### 2.7. Perioperative Data, Histological Findings and Peripheral Blood Cell Index

Data on patient demographics including age, sex, eastern cooperative oncology group-performance status (ECOG-PS), blood examinations, tumor markers, and clinical stage and resectability were measured before and after CRT. Operative findings and postoperative outcomes were also recorded. Final tumor staging was determined histologically according to the JPS 7th edition.

The PNI, an immune-nutritional marker that we previously reported [21], was calculated according to Onodera’s definition as follows: 10 × serum albumin (g/dL) + 0.005 × total lymphocyte count (/µL) [32]. Peripheral blood cell indices were defined as ratios calculated from neutrophil, lymphocyte, and monocyte counts. The neutrophil-lymphocyte-to-monocyte ratio (NL/M) was calculated as (neutrophil count [/µL] × lymphocyte count [/µL])/monocyte count [/µL], and the lymphocyte-monocyte-to-neutrophil ratio (LM/N) was calculated as (lymphocyte count [/µL] × monocyte count [/µL])/neutrophil count [/µL]. The NLR was calculated as neutrophil count/lymphocyte count [22], and the LMR as lymphocyte count/monocyte count [23]. The SIRI was calculated as (neutrophil count × monocyte count)/lymphocyte count [24]. These peripheral blood cell indices and PNI were measured as host-related factors.

### 2.8. Statistical Analyses

Continuous variables are presented as medians with interquartile ranges (IQR) and were compared using the Mann–Whitney U test for two-group comparisons and the Kruskal–Wallis test for comparisons among four groups. Categorical variables were compared using the chi-square test or Fisher’s exact test, as appropriate. Overall survival (OS) was calculated from the date of initiation of neoadjuvant CRT to the date of death from any cause or the last encounter. Patients who were alive at the last encounter were censored on that date. Among patients who underwent surgical resection, postoperative recurrence-free survival (RFS) was calculated from the date of surgery to the date of first recurrence or death from any cause, whichever occurred first. Patients who were alive without recurrence at the last follow-up were censored on that date. Survival curves were estimated using the Kaplan–Meier method and compared using the log-rank test. Patients with postoperative mortality (deaths occurring within 90 days after surgery) were included in both the survival analyses and Cox proportional hazards regression analyses.

Among all BR patients who underwent surgical resection after CRT (*n* = 100), prognostic factors for OS were evaluated using Cox proportional hazards regression analyses. Six of the resected patients lacked post-CRT blood cell counts and were excluded from this analysis for prognosis. For regression analyses, NL/M values were divided by 1000, and CA19-9 levels were natural log-transformed due to skewed distribution. Four variables were included as candidates in the multivariable Cox regression analysis: post-CRT CA19-9, post-CRT NL/M, margin-positive resection, and Evans grade ≥III. These variables were selected based on their clinical relevance, with reference to the results of univariable analyses. CA19-9 was included because of its clinical importance, although it was not significant in the univariable analysis. Forward stepwise selection based on the likelihood ratio was used to identify prognostic factors, and the four variables were also entered simultaneously (enter method) for comparison. Among the peripheral blood cell indices including NLR, LMR, and SIRI, the NL/M, which showed the strongest association with survival in univariable analysis among the indices examined (Table S1), was selected as the representative index and entered the multivariable analysis. This selection was exploratory, and NL/M was not prespecified as the primary biomarker. Hazard ratio (HR) and 95% confidence interval (CI) were calculated. A sensitivity analysis after excluding one patient with an extreme NL/M value was also performed. The optimal cutoff value for NL/M was determined as 3.785 × 10^3^ using Cutoff Finder (https://mybiosoftware.com/tag/cutoff-finder, accessed on 28 August 2026), a web-based application for cutoff point optimization in biomarker analysis, by minimizing the *p* value of the log-rank test for OS (Figure S2) [33].

For surviving patients who had not attended the outpatient clinic for more than 6 months, survival status was confirmed by telephone, and no patients were lost to follow-up. Statistical analyses were performed using SPSS Statistics version 29 (IBM Corp., Armonk, NY, USA). A two-sided *p* value < 0.05 was considered statistically significant.

## 3. Results

### 3.1. Patient Characteristics

A total of 206 patients were included in the analysis: 49 with R-PDAC, 24 with biological BR, 43 with BR-PV, and 90 with BR-A. As shown in Table 1, no significant differences were observed among the four groups in age, sex, ECOG-PS, the prevalence of poorly controlled diabetes mellitus (HbA1c > 8.0%), pretreatment CEA levels, or pretreatment PNI. In contrast, tumor location differed significantly among the groups (*p* = 0.025), with pancreatic head tumors being more frequent in the anatomical BR groups. Pretreatment CA19-9 levels were significantly higher in the biological BR group than in the other groups (*p* < 0.001), whereas tumor size before CRT was larger in the anatomical BR groups (*p* < 0.001). Similar differences were observed after CRT, with higher CA19-9 levels in the biological BR group and larger tumor sizes in the anatomical BR groups (both *p* < 0.001). The resection rates differed significantly among the groups (*p* = 0.004). The resection rate of biological BR was 83.3% and similar to that of R (85.7%). Anatomical BR had lower resection rate (69.8% for BR-PV and 55.6% for BR-A), compared to biological BR.

### 3.2. Long-Term Oncological Outcomes

**Figure 2:**
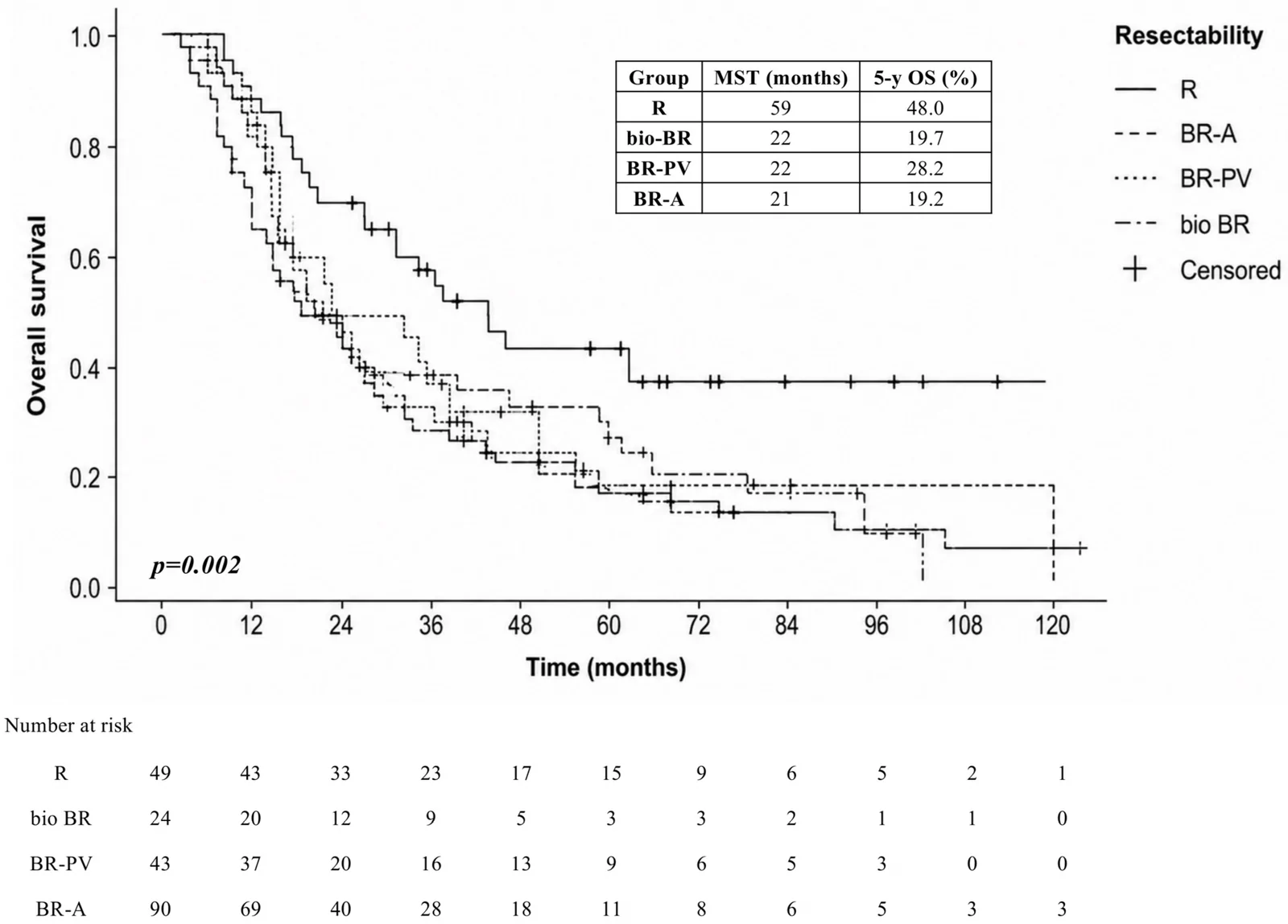
Overall survival according to resectability before GS-CRT. Kaplan–Meier curves of overall survival according to resectability before GS-CRT in patients with pancreatic cancer who received GS-CRT as neoadjuvant treatment. Patients were classified as resectable (R), borderline resectable with arterial involvement (BR-A), borderline resectable with portal vein involvement (BR-PV), or biologically borderline resectable (bio BR). In Cox regression analysis, the HRs for OS were 0.908 (95% CI, 0.598–1.378) for BR-PV and 0.858 (95% CI, 0.518–1.423) for biological BR, using BR-A as the reference group. Abbreviations: bio BR, biologically borderline resectable; BR-A, borderline resectable with arterial involvement; BR-PV, borderline resectable with portal vein involvement; OS, overall survival; R, resectable.

The median OS was 59 months in patients with R disease, 22 months in those with biological BR, 22 months in those with BR-PV, and 21 months in those with BR-A. The corresponding 5-year OS rates were 48.0%, 19.7%, 28.2%, and 19.2%, respectively. In Figure 2, OS differed significantly among the four groups (*p* = 0.002), with patients with R disease showing more favorable survival than those with BR disease. In contrast, OS did not differ significantly across the three BR subgroups (overall log-rank test, *p* = 0.760). In Cox regression analysis among the 157 patients with BR-PDAC, the HRs for OS were 0.908 (95% CI, 0.598–1.378) for BR-PV and 0.858 (95% CI, 0.518–1.423) for biological BR, using BR-A as the reference group.

**Figure 3:**
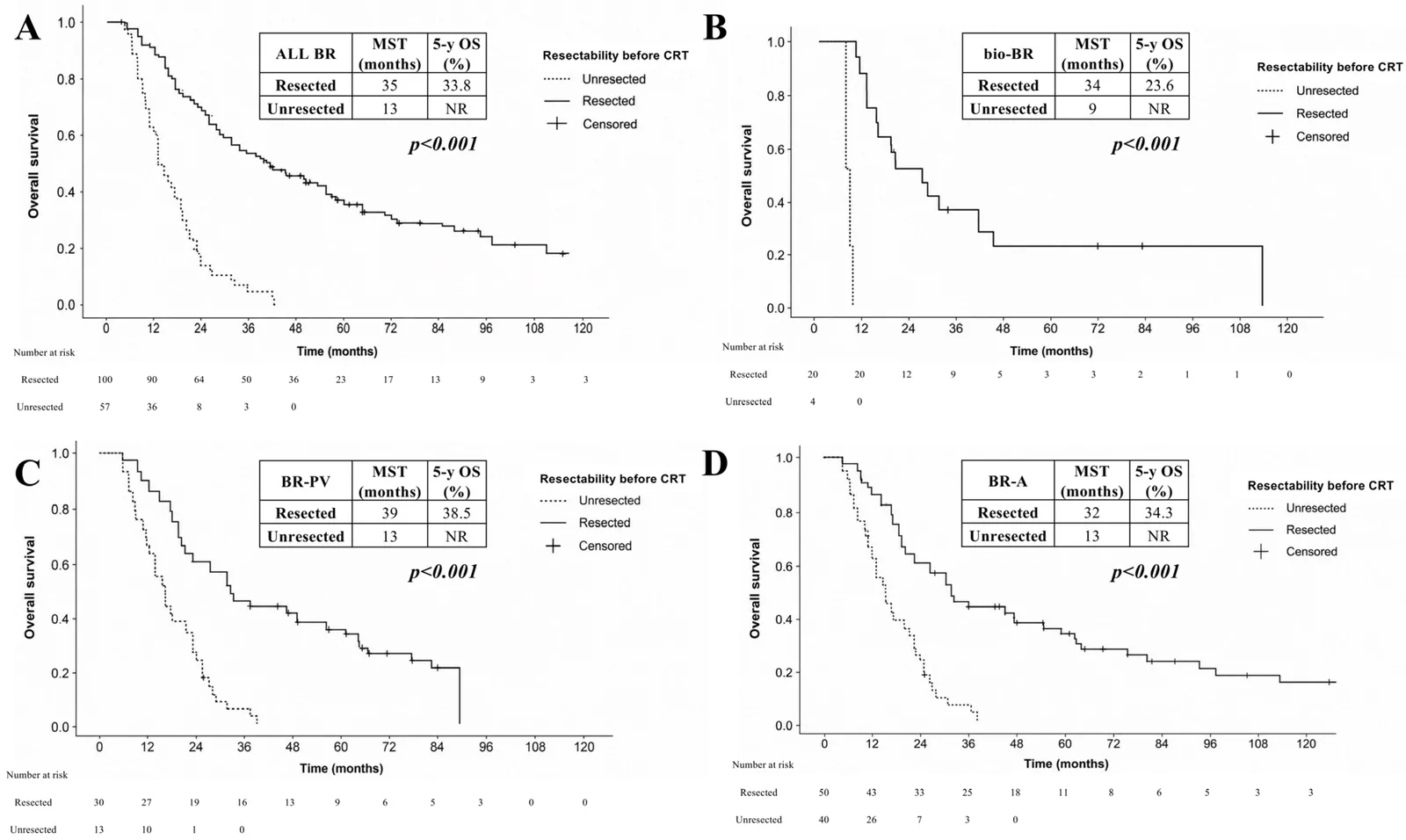
Overall survival according to resection status after neoadjuvant GS-CRT. Kaplan–Meier curves of overall survival according to resection status in patients with borderline resectable pancreatic cancer who received neoadjuvant GS-CRT. (**A**) All patients with borderline resectable pancreatic cancer, (**B**) bio BR, (**C**) BR-PV, and (**D**) BR-A. Solid and dotted lines indicate resected and unresected patients, respectively. Abbreviations: bio BR, biologically borderline resectable; BR-A, borderline resectable pancreatic cancer with arterial involvement; BR-PV, borderline resectable pancreatic cancer with portal vein involvement; NR, not reached.

Among patients with BR-PDAC, surgical resection after CRT was associated with longer OS (median OS, 35 vs. 13 months in resected and unresected patients, respectively). The 3- and 5-year OS rates in the resected group were 50.0% and 33.8%, respectively, whereas the 3-year OS rate in the unresected group was 6.4% and no patient survived beyond 5 years (*p* < 0.001, Figure 3A). This survival difference was consistently observed within each BR subgroup. Median OS was 34 versus 9 months in biological BR, 39 versus 13 months in BR-PV, and 32 versus 13 months in BR-A for resected versus non-resected patients, respectively (all *p* < 0.001). To account for differences in background characteristics and immortal time bias, a time-dependent Cox regression analysis with post-CRT reassessment as the time origin was performed in all 157 patients with BR-PDAC (128 deaths). Surgical resection remained significantly associated with a lower hazard of death in both the unadjusted analysis (HR 0.263, 95% CI, 0.177–0.392, *p* < 0.001) and the multivariable analysis adjusted for age, post-CRT CA19-9, post-CRT tumor size, and BR classification (adjusted HR 0.270, 95% CI, 0.172–0.425, *p* < 0.001; Table S2).

**Figure 4:**
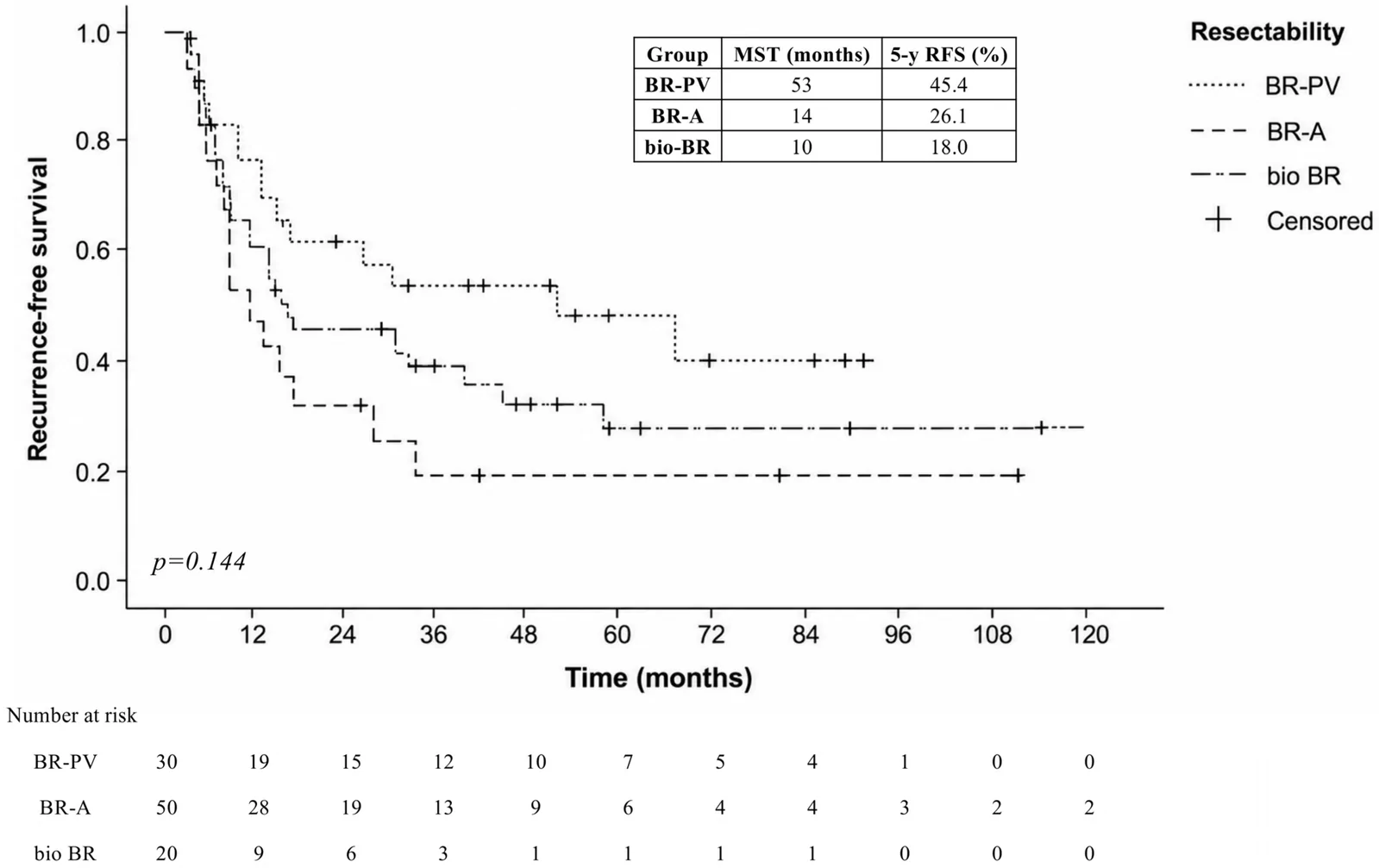
Postoperative recurrence-free survival according to borderline resectability before GS-CRT. Kaplan–Meier curves of postoperative recurrence-free survival in patients with BR pancreatic cancer who underwent surgical resection following neoadjuvant GS-CRT. Patients were classified before GS-CRT as borderline resectable with arterial involvement (BR-A), borderline resectable with portal vein involvement (BR-PV), or biologically borderline resectable (bio BR). Abbreviations: bio BR, biologically borderline resectable; BR-A, borderline resectable with arterial involvement; BR-PV, borderline resectable with portal vein involvement.

Among patients with BR-PDAC who underwent surgical resection, postoperative recurrence-free survival (RFS) was also evaluated according to the initial resectability category. The median RFS was 10 months for biological BR, 53 months for BR-PV, and 14 months for BR-A, with corresponding 5-year RFS rates of 18.0%, 45.4%, and 26.1%, respectively. Postoperative RFS did not differ significantly across the three groups (overall log-rank test, *p* = 0.144; Figure 4). Among patients who developed recurrence after resection, the most common sites of first recurrence were the liver and lung (25.8% each), followed by the peritoneum (19.4%), local recurrence (11.3%), other sites (8.1%), and simultaneous recurrence at multiple sites (9.7%; Table S3). Recurrence sites could not be determined in three patients who received treatment at other institutions.

Median follow-up among surviving patients: 62.5 months (IQR, 40–84 months). The longest follow-up period was 164 months.

### 3.3. Clinicopathological Characteristics of BR-PDAC Patients According to Surgical Resection

Patients who underwent resection were younger than those who did not (median, 68 vs. 71 years, *p* = 0.034), whereas sex, ECOG performance status, and PNI (before CRT) were comparable (Table 2). Before GS-CRT, tumor markers and tumor size tended to be lower in the resected group without reaching significance (CA19-9, 348.0 vs. 603.0 U/mL, *p* = 0.064; tumor size, 30.4 vs. 32.7 mm, *p* = 0.090). After GS-CRT, tumor size decreased in the resected group but not in the unresected group (26.4 vs. 32.6 mm, *p* < 0.001). CA19-9 decreased in both groups but remained higher in the unresected group (48.0 vs. 153.9 U/mL, *p* < 0.001). The post-CRT PNI was also higher in the resected group (41.9 vs. 40.0, *p* = 0.014).

### 3.4. Prognostic Factors Among Patients with BR Disease Who Underwent Surgical Resection

We subsequently evaluated prognostic factors for OS among patients with BR-PDAC who underwent surgical resection after CRT. Among the 94 patients included in the Cox regression analysis, 68 deaths were observed. The results of the univariable analysis are shown in Table S4. In the multivariable analysis using forward stepwise selection, only post-CRT NL/M was retained in the final model (HR 1.020, 95% CI 1.005–1.035, *p* = 0.009), while NL/M was not significant when the four variables were entered simultaneously (HR 1.013, 95% CI 0.996–1.030, *p* = 0.137, Table 3). A sensitivity analysis was performed after excluding one patient with an extreme NL/M value of 106.6 × 10^3^ (neutrophil count 2600/µL, lymphocyte count 410/µL, and monocyte count 10/µL, Figure S2). Post-CRT NL/M was significantly associated with OS in the enter-method model (HR 1.077, 95% CI 1.027–1.130, *p* = 0.002, Table 3). Accordingly, the independent prognostic value of post-CRT NL/M should be regarded as uncertain, because its significance depended on the analysis methods.

**Figure 5:**
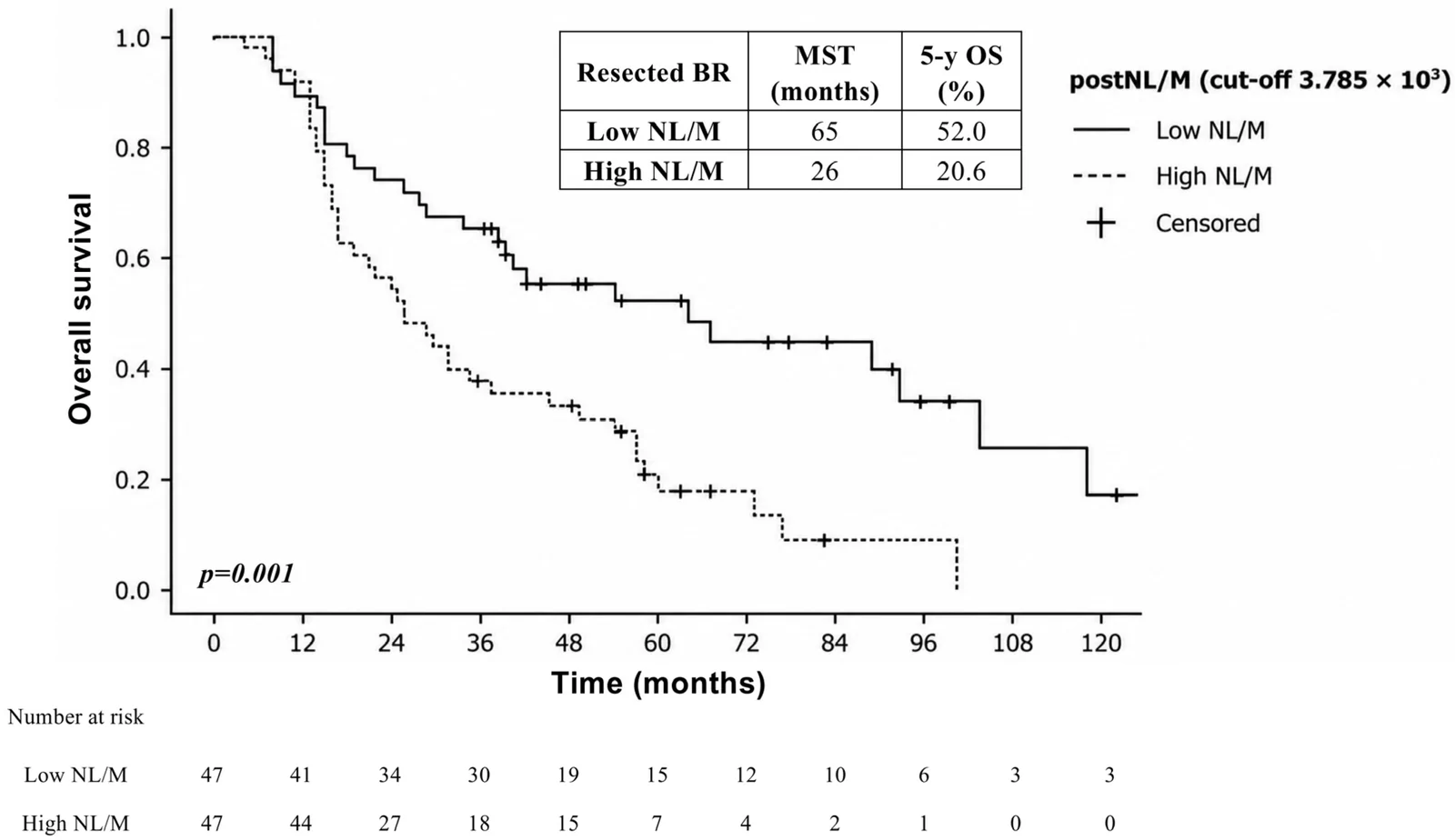
Overall survival according to post-CRT NL/M in patients who underwent resection after GS-CRT. Kaplan–Meier curves of overall survival according to post-CRT NL/M in patients with borderline resectable pancreatic cancer who underwent resection after GS-CRT. Patients were divided into low and high NL/M groups using a cutoff value of 3.785 × 10^3^. Solid and dotted lines indicate the low and high NL/M groups, respectively. Abbreviations: NL/M, neutrophil-lymphocyte-to-monocyte ratio.

The cutoff value of post-CRT NL/M for OS was determined to be 3.785 × 10^3^ using the Cutoff Finder application. Patients with a high post-CRT NL/M had significantly poorer prognosis than those with a low post-CRT NL/M (median OS, 26 vs. 65 months). The 3- and 5-year OS rates were 37.5% and 20.6% in the high NL/M group and 65.2% and 52.0% in the low NL/M group, respectively (*p* = 0.001, Figure 5).

### 3.5. Clinicopathological Characteristics of Resected Patients with BR-PDAC According to Post-CRT NL/M

Before CRT, the low and high NL/M groups were comparable in age, sex, tumor location, resectability category, ECOG performance status, tumor markers, and tumor size (Table 4). The lymphocyte count was higher in the high-NL/M group (1540 vs. 1309/µL, *p* = 0.015), whereas neutrophil and monocyte counts did not differ. After CRT, the high-NL/M group had a higher lymphocyte count (900 vs. 500/µL, *p* < 0.001) and a lower monocyte count (450 vs. 580/µL, *p* < 0.001). Tumor markers and tumor size remained comparable. The incidence of CTCAE Grade ≥3 adverse events did not differ significantly between the high- and low-NL/M groups—(37.5% vs. 47.8%, *p* = 0.311). The operation time was longer in the high-NL/M group (*p* = 0.016), whereas blood loss, the rates of portal vein and arterial resection, Clavien–Dindo grade ≥III complications were comparable. The proportion of patients who received adjuvant chemotherapy for ≥3 months did not differ. Pathologically, T and N categories, local extension factors, and Evans grade were comparable, whereas margin positive resection was observed in the high-NL/M group (14.6% vs. 0%, *p* = 0.006).

## 4. Discussion

To our knowledge, the present study is one of the few studies to evaluate long-term oncological outcomes in patients with anatomical or biological BR-PDAC treated with a consistent neoadjuvant CRT regimen. The median follow-up period in the present cohort was 62.5 months with the longest follow-up extending beyond 13 years. Notably, no statistically significant difference in survival was observed between patients with biological BR and those with anatomical BR. Surgical resection was associated with longer survival across all BR subtypes. We further found that the post-CRT NL/M, a peripheral blood cell-based index, was associated with survival after surgery. These findings provide fundamental information for understanding the long-term oncological outcomes of BR-PDAC.

The distinction between anatomical and biological BR disease is clinically important because the factors defining BR differ substantially between these categories. In the present cohort, patients with anatomical BR disease (BR-A and BR-PV) more frequently had tumors located in the pancreatic head and had larger tumors both before and after CRT than those with R or biological BR disease. These findings likely reflect, at least in part, the anatomical criteria used to define BR disease, which are based on the extent of tumor involvement of major peripancreatic vessels. In the present study, biological BR disease was defined based on the biological criteria proposed by the international consensus, using a pretreatment CA19-9 level ≥500 U/mL. The pre-CRT CA19-9 level was highest in the biological BR group (median 1234.7 U/mL), compared with that in the other groups (77.5 in R, 173.0 in BR-PV, 223.5 in BR-A). After CRT, CA19-9 decreased in all groups, but the biological BR group still had the highest level of 141.2 U/mL. From these interesting findings, unfavorable tumor biology in the biological BR group appears to persist after neoadjuvant CRT. The resection rates were 85.7% in patients with R, 83.3% in those with biological BR, 69.8% in BR-PV, and 55.6% in BR-A. The relatively high resection rate in biological BR likely reflects its anatomical resectability.

Compared with patients with resectable PDAC, those with biological BR did not achieve similarly favorable long-term outcomes despite a comparable resection rate. Their survival did not differ significantly from that of patients with anatomical BR (Figure 2). This discrepancy between anatomical resectability and oncological outcome suggests that anatomical assessment alone may underestimate the oncological risk of biological BR. Across all BR subtypes, surgical resection after CRT was associated with longer OS (Figure 3). Among resected patients, the median overall survival was 34 months for biological BR, 39 months for BR-PV, and 32 months for BR-A, with 5-year survival rates of 23.6%, 38.5%, and 34.3%, respectively. Three patients had 10 years or more survival. In contrast, no patient who did not undergo resection survived for more than 5 years, regardless of resectability categories. Although the number of non-resected patients was limited, particularly in the biological BR group, these findings are consistent with previous studies [34,35] showing favorable long-term outcomes among patients who were able to proceed to surgical resection after neoadjuvant treatment. To account for selection bias and immortal time bias, which may confound the association between resection and survival, we additionally performed a time-dependent Cox regression analysis adjusted for age, post-CRT CA19-9, post-CRT tumor size, and BR classification. Surgical resection remained significantly associated with longer OS (adjusted HR 0.270, 95% CI 0.172–0.425, *p* < 0.001, Table S2). Regarding postoperative recurrence, median RFS was shortest in biological BR (10 months). The differences among the three groups tended to widen for RFS compared with OS (Figure 4). However, these findings are exploratory because of the small number of patients. Distant recurrence, mainly in the liver and lung, was more frequent than local recurrence (11.3%) (Table S3), suggesting that systemic disease control, rather than local control, is the main issue after resection in BR-PDAC, including biological BR.

Despite the favorable outcomes observed among resected patients, determining which patients should proceed to surgery after neoadjuvant treatment remains challenging. Among the factors considered in post-treatment assessment, changes in serum CA19-9 levels are clinically important. Accumulating evidence suggests that normalization or a substantial reduction in CA19-9 during neoadjuvant treatment is associated with improved oncological outcomes after resection and favorable pathological response. However, the optimal definition of CA19-9 response remains unclear. Furthermore, as neoadjuvant therapy affects the host systemically, CA19-9 dynamics, which reflect tumor biology, alone may be insufficient to assess host factors such as immune status, when determining surgical candidacy after CRT. Recent expert consensus has emphasized that assessment for surgical resection after neoadjuvant therapy should be individualized and should integrate multiple factors, including anatomical resectability, CA19-9 response, patient comorbidities, and performance status [18,36,37].

We focused on systemic inflammatory and immune markers derived from a peripheral blood cell-based index as host-related factors complementary to CA19-9. The NLR [22], the LMR [23], and the SIRI [24], have been reported to be associated with prognosis in patients with PDAC. Among patients who underwent surgical resection after CRT, we identified post-CRT NL/M as a prognostic factor. Patients with a high post-CRT NL/M had significantly poorer survival after resection (5-year survival rates of 20.6% versus 52.0%, *p* = 0.001, Figure 5). Patients with a high post-CRT NL/M had longer operative times and a higher rate of margin-positive resection than those with a low NL/M. Persistent inflammation and fibrosis may contribute to increased surgical difficulty and a higher risk of margin-positive resection. Among the conventional peripheral blood cell-based indices (NLR, LMR, and SIRI), only the post-CRT LMR was significantly associated with OS in the univariable analysis (HR 1.046, *p* = 0.034), although its association was weaker than that of NL/M (HR 1.020, *p* = 0.009) (Table S1). CA19-9 and PNI were not significantly associated with OS (Table S4). In our previous study [21], only 25% of patients had BR disease, the PNI was prognostic in the overall cohort but not in the BR subgroup, consistent with the present findings. The NL/M may capture host characteristics more relevant to this population than PNI. The clinical utility of NL/M lies in the timing of its assessment: when measured after CRT, it enables postoperative prognosis to be stratified at the completion of neoadjuvant therapy from the perspective of host factors.

Peripheral blood cell-based indices are generally associated with favorable outcomes when lymphocyte counts are higher and monocyte counts are lower [23]. In our study, however, a high post-CRT NL/M, reflecting a higher lymphocyte and a lower monocyte count, was associated with poorer survival. A similar reversal after CRT has been reported in pancreatic cancer, in which the favorable direction of the monocyte count differed between the intratreatment and post-treatment time points [38]. Post-CRT blood cell counts may therefore not follow the conventional interpretation. The mechanism is unclear, and the following explanations remain hypothetical. First, regarding radiation-induced lymphopenia, lymphocytes are highly radiosensitive and their recovery varies among patients [39,40]. A higher count may thus reflect the recovery pattern rather than antitumor immunity. Second, regarding T-cell subsets, the higher lymphocyte count may include dysfunctional or regulatory T cells, which were not assessed in this study. Third, regarding bone marrow suppression, monocyte counts are usually maintained or increased during and after CRT [39,41]. A persistently low monocyte count may therefore indicate impaired hematopoietic recovery. Monocyte recruitment into the tumor [42], tumor-associated macrophages [43], and treatment-related changes in monocyte function [44,45] may also contribute. Further studies with immune cell subset analysis [41] are required.

In the clinical setting, post-CRT NL/M may have potential as a prognostic marker complementary to CA19-9. Even when tumors remain anatomically resectable after CRT, CA19-9 may not fully reflect tumor biology because of tumor heterogeneity or concomitant cholangitis. To explore the potential complementary prognostic value of post-CRT NL/M and CA19-9, we further evaluated the association between NL/M and OS in patients with post-CRT CA19-9 levels ≤100 U/mL. A low post-CRT NL/M was still associated with longer survival than a high NL/M (Low Group, median OS 94 months, 5-y OS 51.0%; high Group, median OS 26 months, 5-y OS 15.5%; log-rank *p* = 0.003). As NL/M can be calculated from routine complete blood counts without additional testing, it may serve as a simple and feasible prognostic marker, particularly when CA19-9 is uninformative due to Lewis antigen negativity or cholangitis. The results regarding NL/M in this study are exploratory and warrant further investigation. Caveats in interpreting these findings are described in detail in the following limitations paragraph.

Several limitations of this study should be acknowledged. First, this was a retrospective, single-center study with a limited sample size. Second, the decision to proceed to surgery was based on clinical judgment, which may have introduced selection bias. To mitigate this bias, we performed a time-dependent Cox analysis adjusted for clinically relevant factors, in which surgical resection remained associated with longer survival (Table S2). Third, factors affecting CA19-9 values could not be completely excluded. Although CA19-9 was measured after improvement of obstructive jaundice, Lewis antigen status was not assessed. These factors may have limited the classification of biological BR-PDAC. In addition, biological BR was defined by CA19-9 alone, without lymph node assessment by PET-CT or biopsy. Fourth, the association between NL/M and survival should be interpreted with caution. NL/M was retained by stepwise selection but not by the enter method, which may reflect that its effect could not be separated from that of margin-positive resection, because margin-positive resection occurred only in the high NL/M group (Table 4). In the four-variable model, a violation of the proportional hazards assumption was suggested for NL/M (time-dependence, *p* = 0.010), and borderline non-linearity was observed (*p* = 0.054). However, these findings were influenced by a single outlier (NL/M = 106.6 × 10^3^, Figure S2). After excluding this patient, NL/M was significantly associated with overall survival (HR 1.077, 95% CI 1.027–1.130, *p* = 0.002, Table 3), and no departure from the proportional hazards assumption (*p* = 0.447) or linearity (*p* = 0.925) was detected. Thus, the prognostic value of post-CRT NL/M remains uncertain. The instability of the model (the small sample size and the influence of a single extreme value) does not fully establish NL/M as an independent prognostic marker. In addition, the cutoff cannot be regarded as generalizable because it was derived and evaluated in the same cohort. Accordingly, the present findings should be considered exploratory. Nevertheless, post-CRT NL/M significantly stratified postoperative survival in BR-PDAC, and further validation in independent cohorts is warranted. Larger studies including unresected patients, a practical cutoff, and external validation are needed, together with studies integrating peripheral blood analyses with pathological and immunological assessments to clarify the underlying mechanisms.

## 5. Conclusions

In conclusion, our long-term data showed that surgical resection after neoadjuvant CRT was associated with longer survival in patients with BR-PDAC, while highlighting the limitations of anatomical resectability alone in assessing oncological risk. The post-CRT NL/M may play an important role for host-related factors in post-CRT risk assessment.

## Figures and Tables

**Table 1:** Baseline characteristics according to resectability classification.

	R (*n* = 49)	bio BR (*n* = 24)	BR-PV (*n* = 43)	BR-A (*n* = 90)	*p* Value
**Clinical characteristics**					
Sex, male/female (%)	27 (55.1)/22 (44.9)	13 (54.2)/11 (45.8)	30 (69.8)/13 (30.2)	57 (63.3)/33 (36.7)	0.430
Age at diagnosis, years	69 [63–72]	70 [66–74]	67 [58–73]	70 [63–75]	0.515
ECOG performance status	37/10/2/0	18/4/2/0	36/5/1/1	72/16/2/0	0.591
Tumor location (Ph/Pb/Pt)	32/8/9	18/2/4	41/2/0	72/10/8	**0.025**
HbA1c > 8.0% (%)	10 (27.0)	6 (30.0)	14 (35.0)	26 (37.1)	0.738
**Tumor status before GS-CRT**					
CA19-9, U/mL	77.5 [29.6–181.5]	1234.7 [709.1–3671.9]	173.0 [71.7–605.0]	223.5 [75.0–714.9]	**<0.001**
CEA, ng/mL	3.5 [2.2–4.4]	6.1 [3.1–8.5]	3.4 [2.3–5.5]	3.8 [2.3–6.4]	0.204
PNI	47.7 [42.5–52.4]	45.9 [43.1–48.8]	49.0 [43.2–52.2]	47.9 [43.6–50.9]	0.745
Tumor size, mm	23.5 [19.4–30.8]	26.8 [18.2–33.6]	30.8 [25.8–35.1]	31.8 [26.9–36.4]	**<0.001**
**Tumor status after GS-CRT**					
CA19-9, U/mL	30.5 [15.4–50.7]	141.2 [69.9–394.3]	31.8 [10.9–232.0]	58.8 [16.7–153.0]	**<0.001**
CEA, ng/mL	2.9 [1.7–4.0]	4.0 [2.5–5.6]	2.7 [1.7–4.9]	3.2 [2.4–5.2]	0.133
PNI	43.8 [40.1–45.7]	44.0 [40.3–46.6]	41.5 [38.4–44.2]	39.4 [36.9–42.7]	0.252
Tumor size, mm	21.8 [16.9–27.8]	23.2 [18.5–27.6]	27.3 [21.0–31.7]	30.3 [23.4–37.8]	**<0.001**
Resection rate, *n* (%)	42 (85.7)	20 (83.3)	30 (69.8)	50 (55.6)	**0.004**

Bold values indicate statistically significant differences (*p* < 0.05). Missing data: HbA1c, *n* = 39. Abbreviations: bio BR, biological borderline resectable; BR-A, borderline resectable with arterial involvement; BR-PV, borderline resectable with portal vein involvement; GS-CRT, gemcitabine plus S-1 chemoradiotherapy; PNI, prognostic nutritional index.

**Table 2:** Comparison of clinical characteristics between resected and unresected patients with BR-PDAC.

	Resected (*n* = 100)	Unresected (*n* = 57)	*p* Value
**Clinical characteristics**			
Sex, male/female (%)	63 (63.0)/37 (37.0)	37 (64.9)/20 (35.1)	0.811
Age at diagnosis, years	68 [60–74]	71 [65–75]	**0.034**
ECOG performance status	79/17/3/1	47/8/2/0	0.837
Tumor location (Ph/Pb/Pt)	85/7/8	46/7/4	0.532
HbA1c > 8.0% (%)	29 (34.1)	17 (37.8)	0.678
**Tumor status before GS-CRT**			
CA19-9, U/mL	348.0 [72.7–869.0]	603.0 [134.4–1261.5]	0.064
CEA, ng/mL	3.6 [2.1–6.3]	4.9 [2.5–7.3]	0.104
PNI	46.6 [43.4–50.2]	47.1 [42.9–51.6]	0.491
Tumor size, mm	30.4 [25.9–35.3]	32.7 [27.0–40.1]	0.090
**Tumor status after GS-CRT**			
CA19-9, U/mL	48.0 [15.8–117.0]	153.9 [46.1–474.2]	**<0.001**
CEA, ng/mL	3.0 [2.0–5.1]	3.9 [2.7–5.8]	**0.043**
PNI	41.9 [39.7–44.8]	40.0 [37.1–43.1]	**0.014**
Tumor size, mm	26.4 [20.6–32.4]	32.6 [26.0–40.3]	**<0.001**

Bold values indicate statistically significant differences (*p* < 0.05). Missing data: HbA1c, *n* = 27. Abbreviations: GS-CRT, gemcitabine plus S-1 chemoradiotherapy; PNI, prognostic nutritional index.

**Table 3:** Multivariable Cox proportional hazards analyses for overall survival in resected patients.

	All Patients (*n* = 94, 68 Deaths)				Patients Without Outlier * (*n* = 93, 67 Deaths)	
	Stepwise (Forward) ^†^		Enter Method ^†^		Enter Method ^†^	
Variables	HR (95% CI)	*p* Value	HR (95% CI)	*p* Value	HR (95% CI)	*p* Value
NL/M, per 1 × 10^3^ increase	1.020 (1.005–1.035)	**0.009**	1.013 (0.996–1.030)	0.137	1.077 (1.027–1.130)	**0.002**
CA19-9, per 1-unit increase in ln[CA19-9]	–	–	0.971 (0.850–1.109)	0.661	1.003 (0.879–1.145)	0.966
Margin positive resection	–	–	1.753 (0.675–4.555)	0.249	1.740 (0.711–4.256)	0.225
Evans grade ≥III	–	–	0.643 (0.376–1.098)	0.105	0.647 (0.377–1.109)	0.113

* One patient with an extreme NL/M value (106.6 × 10^3^; neutrophil count 2600/µL, lymphocyte count 410/µL, and monocyte count 10/µL) was excluded. ^†^ Four variables were included as candidates in the multivariable Cox regression analysis. Forward stepwise selection based on the likelihood ratio and enter method were performed. Bold values indicate statistically significant differences (*p* < 0.05). Abbreviations: CA19-9, carbohydrate antigen 19-9; CI, confidence interval; HR, hazard ratio; NL/M, neutrophil-lymphocyte-to-monocyte ratio.

**Table 4:** Clinicopathological characteristics according to post-CRT NL/M in resected patients.

	Low NL/M (*n* = 46)	High NL/M (*n* = 48)	*p* Value
**Before CRT**			
Age, years	69 [59–74]	68 [60–72]	0.542
Sex, male/female (%)	31 (67.4)/15 (32.6)	29 (60.4)/19 (39.6)	0.482
Tumor location, Ph/Pb/Pt	37/3/6	44/3/1	0.126
Resectability, BR-A/BR-PV/bio BR	27/12/7	20/16/12	0.236
ECOG PS, 0/1/2/3	38/7/1/0	36/9/2/1	0.660
HbA1c > 8% (%)	12 (28.6)	17 (39.5)	0.286
CA19-9, U/mL	360.9 [80.2–958.2]	208.0 [67.7–626.6]	0.232
CEA, ng/mL	3.1 [1.8–7.7]	3.6 [2.3–6.3]	0.826
Neutrophil count,/µL	3730 [2415–4455]	3280 [2715–4080]	0.395
Lymphocyte count,/µL	1309 [925–1777]	1540 [1285–1940]	**0.015**
Monocyte count,/µL	420 [320–530]	420 [300–535]	0.553
Tumor size, mm	31.0 [26.0–36.5]	30.1 [26.2–33.5]	0.620
**After CRT**			
CA19-9, U/mL	53.0 [16.1–125.9]	37.3 [15.0–79.8]	0.235
CEA, ng/mL	2.5 [1.8–5.2]	3.1 [2.5–4.9]	0.113
Neutrophil count,/µL	2698 [2215–3240]	2900 [2275–3940]	0.204
Lymphocyte count,/µL	500 [355–615]	900 [624–1322]	**<0.001**
Monocyte count,/µL	580 [463–770]	450 [318–575]	**<0.001**
Tumor size, mm	27.2 [21.2–33.8]	25.7 [19.6–30.0]	0.344
CTCAE Grade ≥3 (%)	22 (47.8)	18 (37.5)	0.311
**Surgical factors and postoperative course**			
Operation time, min	472 [416.5–559.5]	540 [494–608]	**0.016**
Blood loss, mL	475 [335–848]	597 [346–1110]	0.294
Portal vein resection (%)	37 (80.4)	41 (85.4)	0.676
Arterial resection (%)	5 (10.9)	1 (2.1)	0.077
Clavien-Dindo grade ≥III, *n* (%)	13 (28.3)	15 (31.3)	0.751
Adjuvant chemotherapy ≥3 months (%)	28 (60.9)	24 (50.0)	0.235
**Pathological factors (%)**			
T category (UICC 8th), 0/1/2/3	1/21/19/5	2/17/28/1	0.167
N category, 0/1/2	36/7/3	31/13/2	0.316
pCH	11 (23.9)	12 (25.0)	0.991
pDU	19 (41.3)	17 (35.4)	0.430
pS	17 (37.0)	14 (29.2)	0.460
pRP	22 (47.8)	17 (35.4)	0.245
pPV	6 (13.0)	12 (25.0)	0.127
pA	2 (4.3)	0 (0)	0.157
pPL	10 (21.7)	13 (27.1)	0.506
pOO	1 (2.2)	0 (0)	0.315
Margin positive resection	0 (0)	7 (14.6)	**0.006**
Evans grade ≥III	21 (45.7)	21 (43.8)	1.000

Bold values indicate statistically significant differences (*p* < 0.05). Missing data: HbA1c, *n* = 9; N category, *n* = 2. Abbreviations: bio BR, biologically borderline resectable; BR-A, borderline resectable with arterial involvement; BR-PV, borderline resectable with portal vein involvement; CA19-9, carbohydrate antigen 19-9; CEA, carcinoembryonic antigen; CRT, chemoradiotherapy; CTCAE, Common Terminology Criteria for Adverse Events; ECOG PS, Eastern Cooperative Oncology Group performance status; HbA1c, hemoglobin A1c; NL/M, neutrophil-lymphocyte-to-monocyte ratio; pA, arterial system invasion; Pb, pancreatic body; pCH, bile duct invasion; pDU, duodenal invasion; Ph, pancreatic head; pOO, invasion of other organs; pPL, extrapancreatic nerve plexus invasion; pPV, portal venous system invasion; pRP, retropancreatic tissue invasion; pS, serosal invasion; Pt, pancreatic tail; UICC, Union for International Cancer Control.

## Data Availability

The data presented in this study are available from the corresponding author upon reasonable request. The data are not publicly available due to privacy and ethical restrictions.

## Supplementary Materials

The following supporting information can be downloaded at: https://www.mdpi.com/article/10.3390/cancers18213521/s1. The Supplementary Materials are provided as a single file (File S1) containing Figure S1: Treatment protocol of gemcitabine/S-1-based neoadjuvant chemoradiotherapy (GS-CRT). Neoadjuvant GS-CRT consisted of concurrent S-1, gemcitabine, and 3D-CRT. S-1 was administered orally at 60 mg/m^2^/day in two divided doses on days 1–21 of a 28-day cycle, and gemcitabine was administered intravenously at 600 mg/m^2^ on days 8 and 22. Radiotherapy was delivered concurrently to a total dose of 50.4 Gy in 25–28 fractions, five fractions per week. The radiation field encompassed the primary tumor, clinically involved regional lymph nodes, and the relevant perivascular regions, with appropriate margins for respiratory motion and setup uncertainty. 238 R/BR patients from November 2011 to December 2022 underwent the GS-CRT therapy. Abbreviation: 3D-CRT, three-dimensional conformal radiotherapy; fr, fraction; GEM, gemcitabine; GS-CRT, gemcitabine/S-1-based chemoradiotherapy; Gy, gray; OP, operation; Table S1: Cox proportional hazards analysis of peripheral blood cell-based indices before and after CRT; Figure S2: Distribution of post-CRT NL/M and its optimal cutoff. Histogram of post-CRT NL/M (× 10^3^) in 94 patients who underwent resection after GS-CRT. Most patients had values below 15 × 10^3^, whereas one patient had an outlier value of 106.6 × 10^3^ (neutrophil count, 2600 cells/µL; lymphocyte count, 410 cells/µL; monocyte count, 10 cells/µL), which was widely separated from the other patients. The optimal cutoff value was determined using Cutoff Finder based on overall survival and was set at 3.785 × 10^3^ (vertical red line). This classified 48 patients (51.1%) into the high NL/M group and 46 (48.9%) into the low NL/M group. Abbreviations: NL/M, neutrophil-lymphocyte-to-monocyte ratio; CRT, chemoradiotherapy; GS-CRT, gemcitabine/S-1-based chemoradiotherapy; Table S2: Time-dependent Cox regression analyses for overall survival in patients with BR pancreatic ductal adenocarcinoma; Table S3: Sites of first recurrence after resection in patients with BR pancreatic ductal adenocarcinoma; and Table S4: Univariable Cox proportional hazards analyses for overall survival in resected patients.
