# Supplementary material for: Long-Term Outcomes of Anatomical and Biological Borderline Resectable Pancreatic Ductal Adenocarcinoma After Gemcitabine/S-1-Based Neoadjuvant Chemoradiotherapy
Source: Cancers (Basel). 2026 Nov 2;18(21):3521. doi: 10.3390/cancers18213521 (PMC13762233; doi:10.3390/cancers18213521)
Supplement: Supplementary file 1 [file cancers-18-03521-s001.zip › cancers-4625504-supplementary.pdf]

cancers

Supplemental Materials

Long-Term Outcomes of Anatomical and Biological Borderline Resectable Pancreatic Ductal Adenocarcinoma After Gemcitabine/S-1-Based Neoadjuvant Chemoradiotherapy

Haruna Komatsubara, Shugo Mizuno, Daisuke Noguchi *, Naohisa Kuriyama, Aoi Hayasaki, Yuki Segi, Kazuyuki Gyoten, Takahiro Ito, Yusuke Iizawa, Takehiro Fujii, Akihiro Tanemura, Yasuhiro Murata and Masashi Kishiwada

*Corresponding author

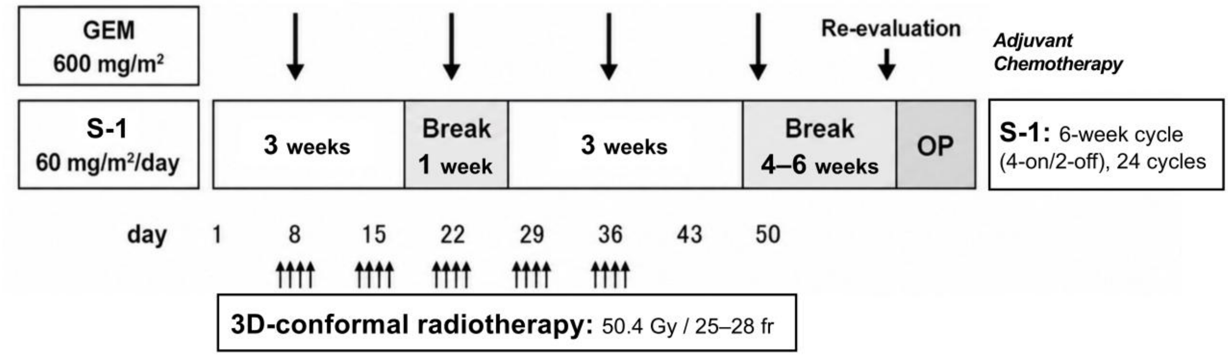

Figure S1. Treatment protocol of gemcitabine/S-1-based neoadjuvant chemoradiotherapy (GS-CRT). Neoadjuvant GS-CRT consisted of concurrent S-1, gemcitabine, and 3D-CRT. S-1 was administered orally at 60 mg/m²/day in two divided doses on days 1–21 of a 28-day cycle, and gemcitabine was administered intravenously at 600 mg/m² on days 8 and 22. Radiotherapy was delivered concurrently to a total dose of 50.4 Gy in 25–28 fractions, five fractions per week. The radiation field encompassed the primary tumor, clinically involved regional lymph nodes, and the relevant perivascular regions, with appropriate margins for respiratory motion and setup uncertainty.238 R/BR patients from November 2011 to December 2022 underwent the GS-CRT therapy. Abbreviation: 3D-CRT, three-dimensional conformal radiotherapy; fr, fraction; GEM, gemcitabine; GS-CRT, gemcitabine/S-1-based chemoradiotherapy; Gy, gray; OP, operation.

Table S1. Cox proportional hazards analysis of peripheral blood cell-based indices before and after CRT.

Variable	Univariable HR (95% CI)	*P* value
Before CRT		
NL/M, per 1 × 10³ increase	0.990 (0.953–1.029)	0.613
NM/L (=SIRI), per 1 × 10³ increase	1.050 (0.827–1.334)	0.689
LM/N, per 1 × 10³ increase	3.462 (0.722–16.606)	0.121
NLR	0.934 (0.780–1.120)	0.462
LMR	0.952 (0.841–1.078)	0.441
After CRT		
NL/M, per 1 × 10³ increase	1.020 (1.005–1.035)	**0.009**
NM/L (=SIRI), per 1 × 10³ increase	0.984 (0.920–1.052)	0.635
LM/N, per 1 × 10³ increase	1.110 (0.294–4.197)	0.877
NLR	0.990 (0.937–1.046)	0.716

 https://doi.org/10.3390/xxxxx

LMR	1.046 (1.004–1.091)	**0.034**

Bold values indicate statistically significant differences ($p < 0.05$). Abbreviations: L, lymphocyte count; LM/N, lymphocyte-monocyte-to-neutrophil ratio; LMR, lymphocyte-to-monocyte ratio; M, monocyte count; N, neutrophil count; NL/M, neutrophil-lymphocyte-to-monocyte ratio; NLR, neutrophil-to-lymphocyte ratio; NM/L, neutrophil-monocyte-to-lymphocyte ratio; SIRI, systemic inflammation response index.

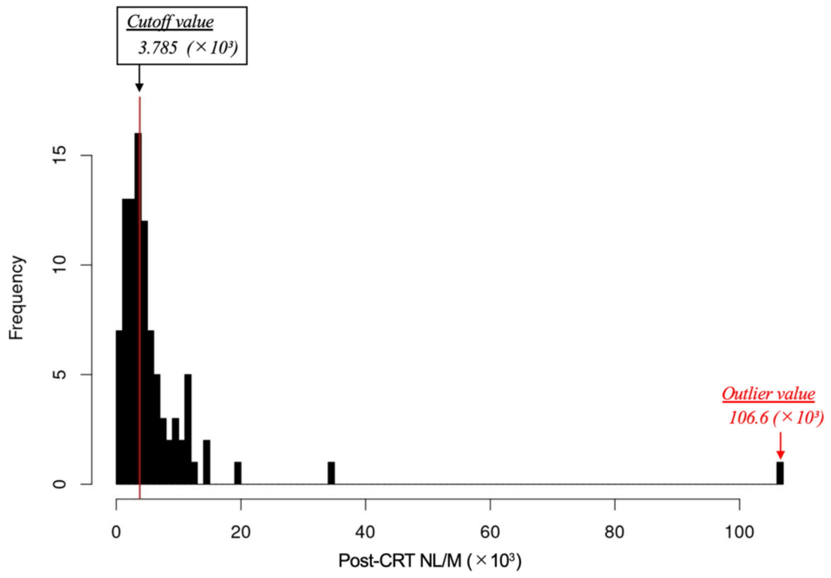

Figure S2. Distribution of post-CRT NL/M and its optimal cutoff. Histogram of post-CRT NL/M (× 10^3) in 94 patients who underwent resection after GS-CRT. Most patients had values below 15 × 10^3, whereas one patient had an outlier value of 106.6 × 10^3 (neutrophil count, 2,600 cells/μL; lymphocyte count, 410 cells/μL; monocyte count, 10 cells/μL), which was widely separated from the other patients. The optimal cutoff value was determined using Cutoff Finder based on overall survival and was set at 3.785 × 10^3 (vertical red line). This classified 48 patients (51.1%) into the high NL/M group and 46 (48.9%) into the low NL/M group. Abbreviations: NL/M, neutrophil-lymphocyte-to-monocyte ratio; CRT, chemoradiotherapy; GS-CRT, gemcitabine/S-1-based chemoradiotherapy.

Table S2. Time-dependent Cox regression analyses for overall survival in patients with BR pancreatic ductal adenocarcinoma.

Variable	Unadjusted HR (95%CI)	*P* value	Adjusted HR (95% CI)	*P* value
Surgical resection (time-dependent)	0.263 (0.177–0.392)	**<0.001**	0.270 (0.172–0.425)	**<0.001**
Before CRT				
Age (year)			1.004 (0.985–1.023)	0.694
BR classification*			1.238 (0.701–2.187)	0.463
After CRT				
CA19-9, per 1-unit increase in ln[CA19-9]			1.017 (0.930–1.112)	0.716
Tumor size (mm)			1.003 (0.985–1.021)	0.761

*BR classification was analyzed as an ordinal variable (BR-A = 0, BR-PV = 1, biological BR = 2). Bold values indicate statistically significant differences ($p < 0.05$). Abbreviations: HR, hazard ratio; CI, confidence interval; CRT, chemoradiotherapy; CA19-9, carbohydrate antigen 19-9; BR, borderline resectable. The analysis included 157 patients (128 deaths), with post-CRT reassessment as the time origin. Surgical resection was modeled as a time-dependent covariate. The multivariable model included age, post-CRT CA19-9, post-CRT tumor size, and BR classification using the enter method.

Table S3. Sites of first recurrence after resection in patients with BR pancreatic ductal adenocarcinoma.

Site of first recurrence	BR-PDAC patients with recurrences (*n* = 62)*	
	**Number**	**%**
Liver	16	25.8
Lung	16	25.8
Peritoneum	12	19.4
Local recurrence	7	11.3
Other sites	5	8.1
Multiple sites (simultaneous)	6	9.7

*Among the 65 patients who developed recurrence after resection, the site of first recurrence was identified in 62 patients and is shown in this table. The site of recurrence in the remaining three patients was unknown because they were treated at other institutions. Percentages were calculated among the 62 patients. Abbreviations: BR-PDAC, borderline resectable pancreatic ductal adenocarcinoma.

Table S4. Univariable Cox proportional hazards analyses for overall survival in resected patients.

**Variable**	**Univariable HR (95% CI)***	***P* value**
Clinical characteristics		
Age	1.008 (0.984–1.032)	0.537
Sex	1.060 (0.659–1.704)	0.810
Primary tumor location	1.037 (0.548–1.962)	0.912
BR classification†	0.920 (0.541–1.565)	0.758
ECOG performance status ≥1	1.253 (0.725–2.165)	0.419
HbA1c >8%	0.726 (0.424–1.242)	0.242
Before GS-CRT		
CA19-9, per 1-unit increase in ln[CA19-9]	0.994 (0.979–1.009)	0.421
CEA, ng/mL	0.995 (0.967–1.023)	0.723
Hemoglobin, g/dL	0.880 (0.759–1.021)	0.092
PNI	1.016 (0.967–1.066)	0.533
NL/M, per 1 × 10^3 increase	0.990 (0.953–1.029)	0.613
Tumor size, mm	0.996 (0.975–1.018)	0.731
Clinical T stage	0.931 (0.197–4.400)	0.928
Clinical N stage	0.819 (0.419–1.601)	0.559
After GS-CRT		
CA19-9, per 1-unit increase in ln[CA19-9]	0.999 (0.892–1.119)	0.985
CEA, ng/mL	1.043 (0.939–1.159)	0.432
Hemoglobin, g/dL	0.895 (0.771–1.038)	0.141
PNI	0.997 (0.950–1.045)	0.889
NL/M, per 1 × 10^3 increase	**1.020 (1.005–1.035)**	**0.009**
Tumor size (mm)	1.012 (0.989–1.036)	0.313
Clinical T stage	0.747 (0.270–2.069)	0.574
Clinical N stage	0.718 (0.288–1.789)	0.477
Operative variables		
Operative time, min	1.001 (1.000–1.003)	0.124
Blood loss, mL	1.000 (1.000–1.000)	0.141
Portal vein resection	1.228 (0.646–2.334)	0.530
Arterial resection	1.973 (0.787–4.951)	0.147
Pathological variables		
ypT stage	1.394 (0.997–1.950)	0.052
ypN stage	1.113 (0.663–1.870)	0.684
Margin positive resection	2.543 (1.134–5.700)	**0.023**
Evans grade ≥III	0.610 (0.378–0.986)	**0.044**

* Total 94 patients, 68 deaths. † BR classification was analyzed as an ordinal variable (BR-A = 0, BR-PV = 1, biological BR = 2). Bold values indicate statistically significant differences ($p < 0.05$). Abbreviations: CA19-9, carbohydrate antigen 19-9; CEA, carcinoembryonic antigen; CI, confidence

interval; CRT, chemoradiotherapy; CTCAE, Common Terminology Criteria for Adverse Events; ECOG PS, Eastern Cooperative Oncology Group performance status; HbA1c, hemoglobin A1c; HR, hazard ratio; JPS, Japan Pancreas Society; NL/M, neutrophil-lymphocyte-to-monocyte ratio; pA, arterial system invasion; Pb, pancreatic body; pCH, bile duct invasion; pDU, duodenal invasion; Ph, pancreatic head; pOO, invasion of other organs; pPL, extrapancreatic nerve plexus invasion; pPV, portal venous system invasion; pRP, retropancreatic tissue invasion; pS, serosal invasion; Pt, pancreatic tail; UICC, Union for International Cancer Control.
